# Corneal Optical Densitometry in the Evaluation of 2-Year Graft Function Following Endothelial Keratoplasty

**DOI:** 10.3390/jcm12041552

**Published:** 2023-02-16

**Authors:** Ilona Piotrowiak-Słupska, Bartłomiej J. Kałużny, Grażyna Malukiewicz

**Affiliations:** Department of Ophthalmology, Division of Ophthalmology and Optometry, Nicolaus Copernicus University, 85-168 Bydgoszcz, Poland

**Keywords:** corneal densitometry, corneal optical density, corneal transparency, Descemet stripping endothelial keratoplasty, Scheimpflug system

## Abstract

This study aimed to assess clinical application of the Scheimpflug corneal tomography for objective evaluation of corneal optical density in eyes undergoing Descemet’s stripping endothelial keratoplasty (DSEK). In this prospective study, 39 pseudophakic eyes with bullous keratopathy were enrolled. All eyes underwent primary DSEK. Ophthalmic examination included best corrected visual acuity (BCVA) measurement, biomicroscopy, Scheimpflug tomography, pachymetry, and endothelial cell count. All measurements were taken preoperatively and within a 2-year follow-up period. Gradual BCVA improvement was observed in all patients. After two years, the mean and median BCVA values were 0.18 logMAR. A decrease in central corneal thickness was noted only during the first 3 months postoperatively and was followed by a gradual increase. Corneal densitometry decreased constantly and most significantly in the first 3 months postoperatively. The consecutive decrease in endothelial cell count of the transplanted cornea was most significant during the first 6 months postoperatively. Six months postoperatively, the strongest correlation (Spearman’s r = −0.41) with BCVA was found for densitometry. This tendency was maintained throughout the entire follow-up period. Corneal densitometry is applicable for objective monitoring of early and late outcomes of endothelial keratoplasty, showing a higher correlation with visual acuity than pachymetry and endothelial cell density.

## 1. Introduction

Corneal transparency determines proper light transmission and is crucial for vision. Any kind of corneal haze is an important pathological symptom requiring further follow-up to enable appropriate therapeutic decision making [1,2,3,4]. Fuchs’ endothelial corneal dystrophy (FECD) is associated with morphological changes in the endothelium, resulting in impaired function leading to corneal edema. Clinically, it contributes to a gradual loss of corneal transparency and a decrease in visual acuity. Supportive therapy includes pharmacological dehydration with hyperosmotic agents (e.g., sodium chloride, glycerol, or mannitol) or more recently introduced Rho-kinase inhibitors; however, its effectiveness is limited to the initial stages of the disease [5,6]. Consecutively with decreasing cell number, spontaneous corneal endothelium decompensation may appear resulting in stromal oedema and clouding. A particular case of the disease is an additional intraoperative cells injury during cataract phacoemulsification. This results in sudden loss of larger number of endothelial cells, often leading to persistent decompensation. More advanced cases require surgical treatment involving endothelial cell transplantation. Several techniques have been introduced, including Descemet stripping endothelial keratoplasty (DSEK), Descemet stripping automated endothelial keratoplasty (DSAEK), ultrathin Descemet stripping automated endothelial keratoplasty (ut-DSAEK), and Descemet’s membrane endothelial keratoplasty (DMEK). In all techniques, Descemet’s membrane with diseased endothelial cells layer is removed and replaced by the tissue obtained from the donor. The transplant involves the endothelium and Descemet’s membrane (DMEK), or additionally posterior stroma (DSEK prepared manually, DSAEK or ut-DSAEK prepared with a microkeratome). Corneal transplantation restores the metabolic function of the endothelial cell layer and subsequently the corneal clarity [7,8,9,10,11,12,13]. Surgical treatment efficacy can be objectively assessed by measuring endothelial cell density (ECD), central corneal thickness (CCT), and corneal transparency (densitometry). So far, routine monitoring has usually been based on subjective evaluation using a slit lamp, pachymetry, and ECD. Scheimpflug corneal tomography with densitometry enables an additional objective quantitative analysis of corneal optical quality.

Densitometry reflects transparency of structures and their ability to transmit light uninterruptedly. Decrease in transparency causes light backscatter and vision deterioration. Backscattered light evaluation allows quantitative assessment of the optical density of the examined tissue. In ophthalmology, it is applied in the analysis of anterior segment structure transparency, mainly the cornea and crystalline lens. Results are shown in grayscale units (GSU) from 0 (transparent) to 100 (opaque) [14].

This study aimed to assess the clinical application of corneal density measured by the Scheimpflug corneal tomography for objective evaluation of DSEK outcomes in eyes operated for pseudophakic bullous keratopathy due to FECD.

## 2. Materials and Methods

**Figure 1:**
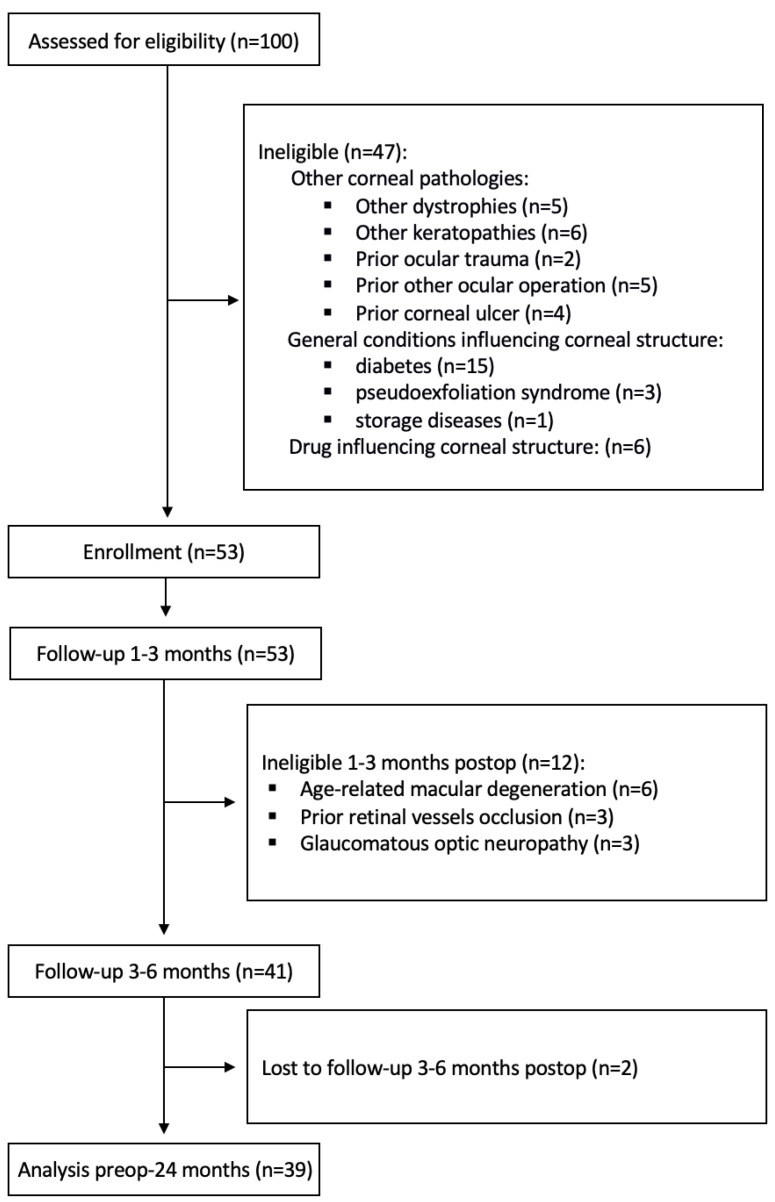
Consort flow-chart. postop, postoperatively.

A total of 100 pseudophakic eyes in 100 patients with bullous keratopathy due to FECD were recruited for this prospective study. All patients were diagnosed with initial stage FECD prior to cataract surgery and decompensated henceforth (inclusion criterion). Exclusion criteria included coexisting pathologies with abnormal corneal transparency (Figure 1). Following the study protocol (Figure 1), data from 39 eyes were finally analyzed (12 men, 27 women; mean age 76 years, range 57–89 years), all Caucasian. Mean time from cataract surgery to corneal decompensation was 32.1 months (range 0–120), mean time from corneal decompensation to DSEK surgery was 10.2 months (range 3–29).

All eyes underwent prior cataract surgery with phacoemulsification and posterior chamber intraocular lens implantation. Subsequently, primary DSEK was conducted due to corneal endothelial decompensation. Subjects were recruited and examined in the Department of Ophthalmology, Nicolaus Copernicus University in Bydgoszcz, Poland after written informed consent was provided by the participants according to the local Bioethics Committee guidelines (KB 605/2013). The study was conducted from 01/2012 to 12/2018.

The complete ophthalmic examination included best corrected visual acuity (BCVA) measurements in Snellen converted to logMAR, biomicroscopy (SL 980 3X, Digital Vision R, Costruzione Strumenti Oftalmici, Firenze, Italy; Sony CCD 1392 × 1040 pixels), Scheimpflug corneal tomography (Pentacam HR, Oculus GmbH, Germany), and assessment of the endothelial cell density (SP 3000P, Topcon, Tokyo, Japan). All measurements were taken preoperatively and within a 2-year follow-up period (1, 3, 6, 12, 18, and 24 months postoperatively).

**Figure 2:**
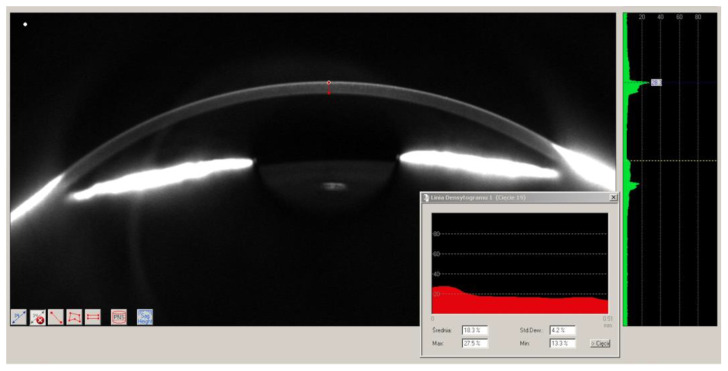
Manual densitometry measurement.

Pentacam (Oculus, Germany) was the first commercially available device using this technology. A digital Scheimpflug corneal tomography provides 50 three-dimensional tomograms of the anterior segment of the eye in different meridians using 475-nm-long monochromatic slit light. Analysis of 138,000 true elevation points enables anterior and posterior surface mapping and pachymetry. Corneal pachymetry and densitometry were evaluated by Pentacam HR using a single measurement of 50 scans in different meridians. A manual analysis of the corneal densitometry was conducted from the outermost epithelium layer to the innermost edges of the endothelial cells on scans in four meridians (180–0, 270–90, 225–45, and 135–315 degrees) performed in the optical axis (corneal center). The results are shown in GSU from 0 (highest transparency, lowest optical density) to 100 (lowest transparency, highest optical density). Due to variability in results according to corneal depth (higher in the anterior layers) and different FECD stages in the study group (more expressed in the inner layers and in the center), we analyzed the mean densitometry over the entire depth of the cornea in the central optical zone in the four meridians (Figure 2).

Pachymetry was assessed using automated CCT measurements by the Pentacam HR. Endothelial cell density was analyzed in the optical axis.

All DSEK operations were performed by the same surgeon (BK), and all parameters were measured and marked by the same person (IPS).

### Statistical Analysis

The normality of the variable distribution was evaluated with the Shapiro–Wilk test, and the statistical characteristics are presented as percentage values and arithmetic means with standard deviations. Error bars in the graphical representation of results indicate standard errors and standard deviations. Levene’s test was used to check the homogeneity of variances in the analyzed samples. The effects of follow-up time on BCVA, CCT, and ECD were evaluated using Friedman’s ANOVA or one-way ANOVA repeated measurements, depending on the distribution characteristics of the analyzed variables. If significant differences were observed by ANOVA, post hoc pair-wise comparisons were performed using Dunn’s or Scheffe’s test, where appropriate. Correlations between variables were analyzed using Spearman’s coefficient model. Percentage proportion data were tested using the chi-square test. Bonferroni correction was applied if appropriate. The level of significance for all tests was set at *p* < 0.05. All calculations were conducted using the STATISTICA 13.0 PL statistical package (StatSoft, Kraków, Poland).

## 3. Results

### 3.1. Variability over Time

**Figure 3:**
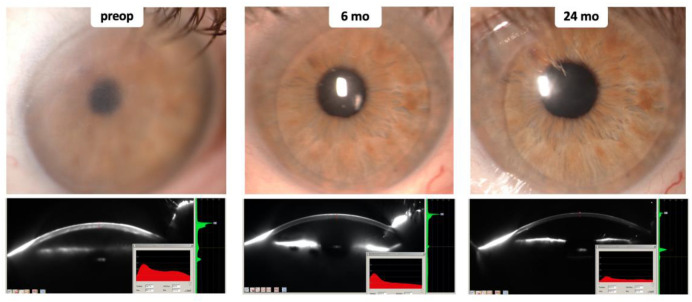
Example of slit-lamp examinations and densitometry readings before DSEK (preoperatively) and 6 and 24 months postoperatively. preop, preoperatively; mo, months; DSEK, Descemet’s stripping endothelial keratoplasty.

**Figure 4:**
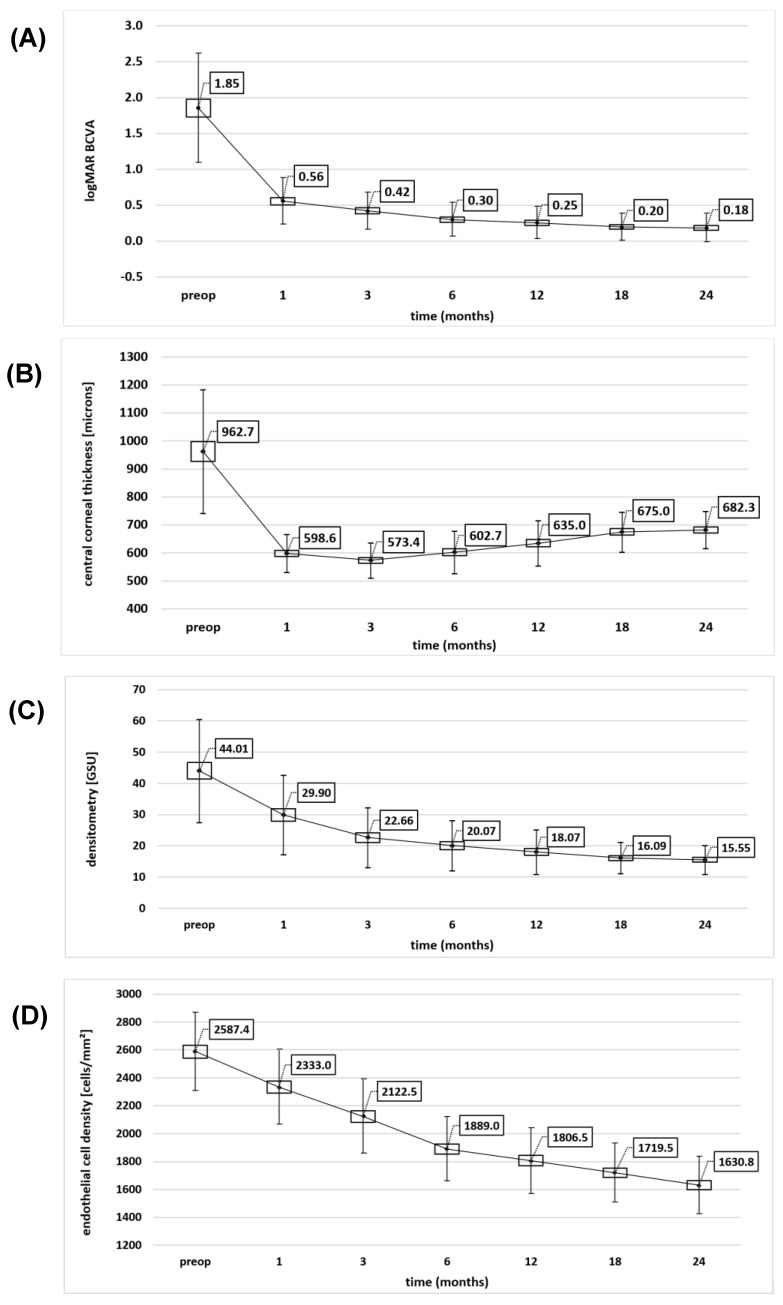
Mean BCVA (**A**), central corneal thickness (**B**), densitometry (**C**), and endothelial cell density (**D**) during the follow-up period. Boxes show the mean ± SE, squares in the boxes represent the mean, and bars represent the mean ± SD. BCVA, best corrected visual acuity; GSU, grayscale units; preop, preoperatively. Precise measurement estimates are provided in Table S1.

Representative pictures of biomicroscopy and densitometry during follow-up period are shown in Figure 3. The mean values of BCVA, CCT, densitometry, and ECD are shown in Figure 4.

#### 3.1.1. BCVA

During the follow-up period, all patients showed gradual improvement in BCVA. The most significant change was observed within the first month postoperatively (*p* < 0.01), subsequently lacking statistically significant differences. After two years, the mean and median BCVA were both 0.18 logMAR (Figure 4A). However, in the 3rd month postoperatively, 4 patients (10.3%) achieved a BCVA of 0.05 logMAR, and after 2 years, this number increased to 10 patients (25.6%).

#### 3.1.2. Pachymetry

A decrease in CCT was observed 1 and 3 months postoperatively (*p* < 0.01 and *p* > 0.05, respectively). Subsequently, a minor, stable increase in corneal thickness was noticed until the end of the follow-up period; however, there was no statistically significant difference between consecutive time points (all *p* > 0.05; Figure 4B).

#### 3.1.3. Densitometry

The mean density decreased during the 2-year follow-up period, showing statistically significant differences in the first 3 months postoperatively (*p* < 0.05; Figure 4C).

#### 3.1.4. Endothelial Cell Density

The most intensive endothelial cell loss was observed in the first 6 months postoperatively (*p* < 0.01). Afterward, further decreases were not significantly different and continued steadily until the end of the follow-up period (Figure 4D). The subsequent loss of transplanted cells did not contribute to endothelial insufficiency (stromal edema and decrease in transparency) in any patient.

The analysis of variance and the post hoc analysis enabled defining time points where no further significant change could be observed (stability of the parameter). Furthermore, the percentage of eyes achieving 24-month mean value or better was calculated for each parameter (equal or less for BCVA, CCT, and densitometry; equal or more for ECD; Table 1).

### 3.2. Functional Stabilization of Vision

**Figure 5:**
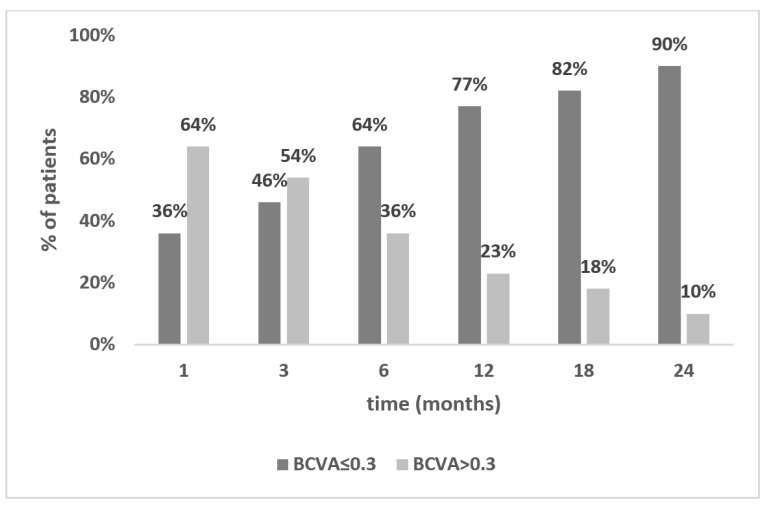
Proportion of patients achieving a BCVA ≤ 0.3 and >0.3 logMAR within the follow-up period. Statistical significance is shown in Table 2. BCVA, best corrected visual acuity.

Stabilization of visual function was established as BCVA ≤ 0.3 logMAR maintained within a further follow-up period. This value was chosen based on the standard World Health Organization definition of visual impairment (mild or no visual impairment for BCVA ≤ 0.5) according to the mean BCVA in the study group. The percentage of eyes achieving BCVA ≤ 0.3 at each time point of the study is shown in Figure 5, with corresponding statistical significance values displayed in Table 2.

Three months after DSEK, the majority of patients still had BCVA values above 0.3 logMAR (54%). By contrast, six months postoperatively, 64% of eyes achieved BCVA ≤ 0.3, and this trend was maintained during the follow-up period.

Comparing consecutive time points of the study, statistical significance between the proportions of eyes below and above BCVA = 0.3 logMAR was noted only between 3 and 6 months postoperatively (*p* = 0.0055; Table 2). The analysis of variance with post hoc analysis showed no statistical difference in BCVA between consecutive time points following 6 months postoperatively. Therefore, we established 6 months as the functional stability time point after DSEK.

**Figure 6:**
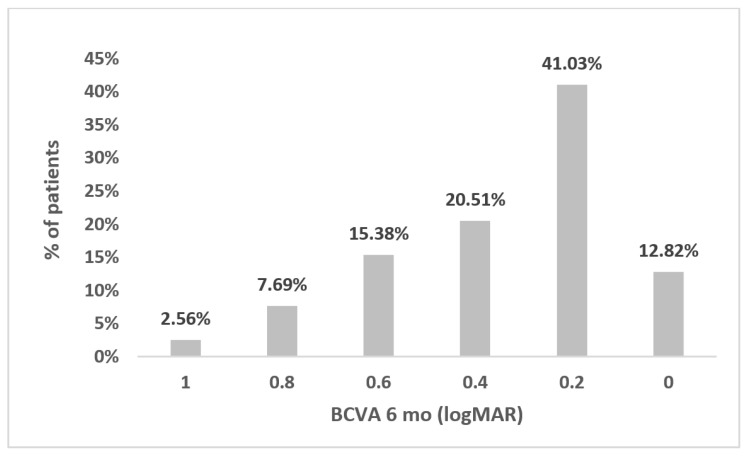
Percentage of BCVA at the time point of visual stabilization (6 months postoperatively). BCVA, best corrected visual acuity; mo, month.

The percentage of eyes achieving the mean BCVA 6 months postoperatively is shown in Figure 6.

### 3.3. Correlation between Parameters at the Stabilization Time Point (6 Months Postoperatively)

Spearman’s correlation coefficients between BCVA, CCT, densitometry, and ECD are shown in Table 3. Among the analyzed parameters, the highest correlation was found for densitometry; however, it remained moderate.

### 3.4. Correlation between Parameters throughout the 2-Year-Follow-Up Period

**Figure 7:**
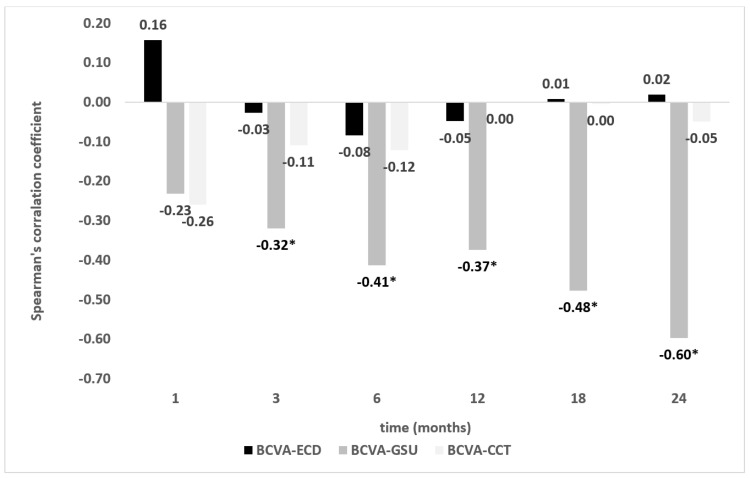
Spearman’s correlation coefficients between BCVA and other analyzed parameters (densitometry, CCT, and ECD) in the postoperative period. * denotes significant differences (*p* < 0.05). BCVA, best corrected visual acuity; CCT, central corneal thickness; ECD, endothelial cell density; GSU, grayscale units.

Densitometry proved to be the only parameter correlated with BCVA in the postoperative period. There was a statistically significant low correlation at 3 and 12 months postoperatively, moderate correlation at 6 and 18 months postoperatively, and high correlation at 2 years postoperatively. Other parameters (CCT and ECD) showed no statistically significant correlation with BCVA during the 24-month follow-up period (Figure 7). Scatterplots of correlations of BCVA with pachymetry and densitometry in the late postoperative period (12–24 months) are shown in Figure S1.

## 4. Discussion

Endothelial keratoplasty aims to regain the regulation of stromal hydration by transplantation of endothelial cells. It enables improvement of corneal clarity resulting in better visual outcomes, expressed as BCVA. Therefore, monitoring graft function based on the evaluation of corneal transparency seems to be a reasonable approach. Optical density measurements have been previously used to assess corneal clarity in infectious keratitis, keratoconus, and following refractive laser surgery and keratoplasty [15,16,17].

According to our data, from 6 months postoperatively most transplanted patients had a postoperative BCVA of ≤0.3 logMAR. In the late postoperative period (from the third month onward), densitometry showed the highest significant correlation with BCVA (r = −0.32 to −0.60; Figure 7). The correlations of CCT and ECD with BCVA were not statistically significant within the study period. In our cohort, physiological values of corneal density (in this age group 16.9 ± 1.9 GSU according to Ní Dhubhghaill et al. [14]) were restored between 12 and 18 months postoperatively. The consecutive decrease in densitometry throughout the study was maintained in all patients, as none of the eyes showed secondary endothelial insufficiency.

The expected clinical follow-up development is associated with improved corneal transparency (low densitometry readings) and good visual acuity (low logMAR BCVA). A high logMAR BCVA (low visual acuity) with low densitometry readings could be caused by irregularities of both the anterior and posterior surfaces of the cornea, as well as the tissue interface, resulting in an increase in higher-order aberrations (HOAs). A high logMAR BCVA (low visual acuity) with high densitometry readings is associated with subepithelial fibrosis or limited edema resorption due to partial primary graft failure, whereas a low logMAR BCVA (good visual acuity) despite high densitometry readings (>50 GSU) could be related to the subjective improvement of comfort due to regression of pain and photophobia [7,11,13].

To date, densitometry has mainly been described for objective quantitative analysis, enabling the comparison of results achieved by different endothelial keratoplasty techniques and their modifications. In DMEK, better visual outcomes with faster recovery are expected owing to a thinner flap and smoother interface, resulting in less light backscattering. By contrast, DSEK and DSAEK enable higher intraoperative safety (lower risk of accidental flap damage) at the cost of irregularities and haze occurring more frequently. Objective analyses of postoperative quantitative parameters reveal the advantages of each technique [18,19,20,21,22,23,24].

Except for the deterioration in light transmission, FECD is often associated with HOAs resulting from stromal architecture disintegration in anterior layers and endothelium with Descemet’s membrane damage in the posterior cornea. HOAs significantly contribute to light backscatter, resulting in higher density. In the study by van Dijk et al., the Pentacam was used for densitometric evaluation, and the results were compared to HOAs in corneas following DMEK. The HOAs at 6 months postoperatively were reduced only in the posterior corneal layers, whereas densitometry readings decreased in all layers. Therefore, density seems to be a more sensitive parameter to monitor cornea conditions postoperatively [25].

Chaurasia et al. examined eyes with FECD and coexisting anterior basement membrane dystrophy or subepithelial fibrosis contributing to lower corneal transparency. All eyes underwent DMEK, in fibrotic eyes additionally coupled with epithelium debridement and mitomycin-C application (MMC). Densitometry of the anterior cornea decreased significantly 6 months postoperatively from 27% to 23% and from 40% to 24% in the DMEK and DMEK + MMC groups, respectively (results are given in % equal GSU) [26]. In our study, we noticed a similar decrease in density (from 44.01 GSU to 20.07 GSU) caused solely by transplanted endothelium function despite using a technique with a higher risk of interface irregularities.

Droutsas et al. conducted a comparative study of DSAEK and DMEK using densitometry over a 2-year follow-up period. Their data proved that the 6-month postoperative density was significantly higher in the DSAEK group; however, after 12 months, the difference between DSAEK and DMEK was not statistically significant. In comparison to the control group consisting of healthy eyes (GSU 18.1 ± 1.3), densitometry readings were significantly higher following DSAEK during the 2-year-follow up, whereas in the DMEK group, no significant difference was first observed at 24 months postoperatively. This confirms the possibility of restoring visual acuity comparable to healthy eyes up to 24 months postoperatively (DMEK 19.2 ± 2.7 GSU, DSEAK 21.2 ± 2.5 GSU) [27]. In our data, the densitometry results of the DSEK group were comparable to those of the cited DSAEK group between 3 and 6 months postoperatively, whereas the results 12 months postoperatively corresponded to those of the control group in their study.

In the study by Arnalich-Montiel et al., densitometry was used to compare primary DSAEK and DSAEK following failed DMEK. Statistically significant differences were observed in biomicroscopy, visual acuity, and densitometry. Lower corneal transparency in DSAEK following DMEK group was due to stromal edema, fibrosis, and interface haze. The 6-month postoperative densitometry readings were 13.71 GSU, 16.72 GSU, and 19.92 GSU for control, primary DSAEK, and DSAEK following DMEK groups, respectively. Densitometry provided objective quantitative analyses confirming subjective slit-lamp observations [28]. In our results, the densitometry readings 6 months postoperatively were similar to those for the DSAEK following DMEK group.

In our cohort, CCT decreased during the first 3 months postoperatively followed by a minor increase. This physiological phenomenon does not alter vision. However, the trend change probably contributes to the low correlation of pachymetry and visual acuity 6 months postoperatively (r = −0.12), maintained during the 24-month follow-up period. It can be assumed that CCT is applicable for monitoring postoperative results until stromal edema resorption (3 months postoperatively), whereas the subsequent CCT increase solely reflects physiological tissue reconstruction [29]. Therefore, pachymetry should not be used for precise detection of stromal edema recurrence, as CCT 6–24 months after DSEK gradually increases. In our study, no secondary clinical graft failure was noted and all grafts remained clear throughout the 24-month study period, so both pachymetry and densitometry could not be assessed for detection of early edema increase. However, it seems justified to presume that a CCT increase due to liquid accumulation will result in a densitometry increase, whereas a CCT increase due to proteoglycans restoration (because of their low molecular weight) should not influence density measured by 475-nm wavelength light, as in the case of our study.

Moreover, discrepancies in pachymetry readings could be due to manual flap preparation causing minor differences between eyes. The Pentacam HR does not enable automatic flap thickness analysis, and the error rate in manual measurements is too high. Therefore, the residual recipient stromal thickness was not analyzed in this study, and only the total CCT could be objectively measured.

In the study by Ivarsen et al., pachymetry was evaluated in correlation to light backscatter and visual acuity at a 2-year follow-up after DSAEK. They demonstrated that CCT decreased in the early postoperative period to increase gradually from 6 months postoperatively, showing a weak correlation with low densitometry readings (Pearson’s r = −0.14; *p* < 0.05). The authors assumed that the initial rapid decrease in CCT is due to edema resorption following the restoration of endothelium function and is accompanied by a decrease in densitometry. By contrast, the gradual increase in CCT in the late postoperative period is caused by a slow accumulation of extracellular matrix, mainly glycosaminoglycans. They restore the proper microarchitecture of collagen fibrils, resulting in a further increase in stromal transparency leading to a decrease in densitometry, even though it is not as intensive as in the early postoperative period [29]. We found a similar tendency in our data; however, confirming this hypothesis would require further examinations at the molecular level.

In our study, a moderate correlation between postoperative densitometry and pachymetry was only noted at 1 month postoperatively (r = 0.43). Six months postoperatively, the correlation coefficient r was 0.03, and oscillated afterward around the zero level; however, changes in the sign of the correlation coefficient (from positive to negative) were observed and may support the stromal restoration theory. Compared to pachymetry, densitometry showed a significantly higher correlation with BCVA throughout the 24-month follow-up period; therefore, it may be a more reliable parameter to monitor corneal function after DSEK.

Progressive endothelial cell loss was similar to that reported in the literature, with 27.23%, 30.69%, and 37.35% at 6, 12, and 24 months postoperatively, respectively [30]. ECD showed no statistically significant correlation with BCVA (r = −0.08), densitometry (r = −0.02), and pachymetry (r = −0.07). It was strictly dependent on the initial transplanted tissue quality, and during the follow-up period, it gradually decreased, as expected.

During the study, no secondary graft failure due to low endothelial cell count was noted that would result in vision deterioration. Presumably, loss of transplanted endothelial cells, regardless of the faster decrease as compared to physiological loss, has no impact on vision and corneal transparency until critically low values are reached. In our study, endothelial cell loss often exceeded the level of decompensation for normal cornea (approximately 500/mm^2^) [29,31,32,33].

### Limitations of the Study

The number of patients was low due to tissue shortage and postoperatively diagnosed ocular pathologies leading to the exclusion of patients. Due to tissue shortage and learning curve, DSEK was the lamellar keratoplasty technique introduced in our center and its results are assessed in this study. Preoperative BCVA was low, therefore it was assessed by regular Snellen charts till 1.85 logMAR, henceforth counting fingers. Furthermore, as compared to DSAEK and DMEK, manual tissue preparation in DSEK might result in flap thickness differences, interface irregularities, scarring, and HOAs. However, all surgical procedures were conducted by one operator, and our results are in agreement with those obtained by other techniques cited in the literature. No intraclass correlation was assessed.

In conclusion, corneal densitometry is applicable for objective monitoring of early and late outcomes of endothelial keratoplasty, showing a higher correlation with visual acuity than pachymetry and endothelial cell density. During the 2-year-follow-up period, progressive endothelial cell loss did not correlate with visual acuity, optical density, or central corneal thickness. Pachymetry changes after DSEK reflect both edema resorption and stromal collagen structure restoration and, therefore, should not be used as an independent follow-up parameter to describe corneal conditions.

## Figures and Tables

**Table 1:** Variability and stabilization of parameters during the follow-up period.

		BCVA	CCT	Densitometry	ECD
Loss of Statistical Significance (*p* > 0.05) of Changes Comparing Subsequent Time Points		6 mo	6 mo	3 mo	12 mo
Mean 24-month value or better	1 mo	7.7%	89.7%	12.8%	100%
	3 mo	20.5%	94.9%	23.1%	100%
	6 mo	38.5%	87.2%	25.6%	84.6%
	12 mo	56.4%	71.8%	46.2%	74.4%
	18 mo	66.7%	48.7%	56.4%	64.1%
	24 mo	66.7%	48.7%	64.1%	46.2%

BCVA, best corrected visual acuity; CCT, central corneal thickness; ECD, endothelial cell density; mo, month.

**Table 2:** Statistical significance of proportion of patients achieving BCVA ≤ 0.3 logMAR referring to >0.3 logMAR within the follow-up period.

	1 mo	3 mo	6 mo	12 mo	18 mo	24 mo
1 mo		>0.051	0.0062	<0.011	<0.013	<0.011
3 mo	>0.051		0.0055	0.0026	<0.014	<0.012
6 mo	0.0062	0.0055		>0.053	0.037	0.0036
12 mo	<0.011	0.0026	>0.053		>0.054	>0.054
18 mo	<0.013	<0.014	0.037	>0.054		>0.053
24 mo	<0.011	<0.012	0.0036	>0.054	>0.053	

mo, month.

**Table 3:** Spearman’s correlation coefficients between BCVA, CCT, densitometry, and ECD 6 months postoperatively.

mean ± SD	Parameter	ECD 6 mo	BCVA 6 mo	GSU 6 mo	CCT 6 mo
1889.00 ± 230.62	ECD 6 mo		−0.08	−0.02	−0.07
0.57 ± 0.26	BCVA 6 mo	−0.08		−0.41 *	−0.12
20.07 ± 8.07	GSU 6 mo	−0.02	−0.41 *		0.03
602.69 ± 75.85	CCT 6 mo	−0.07	−0.12	0.03	

* denotes significant differences (*p* < 0.05). BCVA, best corrected visual acuity; CCT, central corneal thickness; ECD, endothelial cell density; GSU, grayscale units; mo, month.

## Data Availability

Not applicable.

## Supplementary Materials

The following supporting information can be downloaded at: https://www.mdpi.com/article/10.3390/jcm12041552/s1.
