# Supplementary material for: Corneal Optical Densitometry in the Evaluation of 2-Year Graft Function Following Endothelial Keratoplasty
Source: J Clin Med. 2023 Feb 16;12(4):1552. doi: 10.3390/jcm12041552 (PMC9963363; doi:10.3390/jcm12041552)
Supplement: Supplementary file 1 [file jcm-12-01552-s001.zip › jcm-2090048-supplementary/Figure S1.pdf]

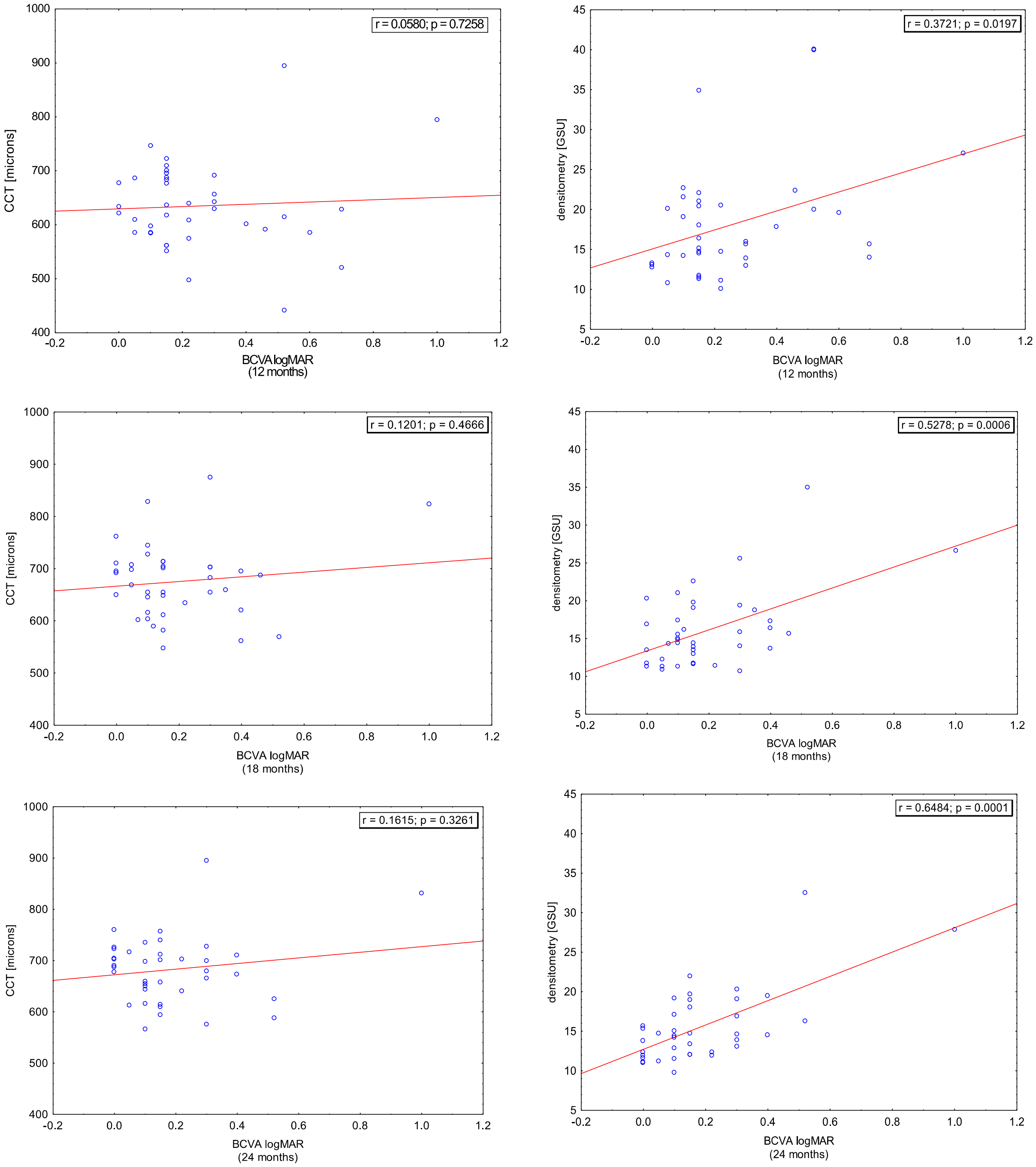

Figure S1. Correlations of BCVA with pachymetry and densitometry in the late postoperative period. BCVA, best corrected visual acuity; CCT, central corneal thickness; GSU, grayscale units.
