# Supplementary material for: Corneal Optical Densitometry in the Evaluation of 2-Year Graft Function Following Endothelial Keratoplasty
Source: J Clin Med. 2023 Feb 16;12(4):1552. doi: 10.3390/jcm12041552 (PMC9963363; doi:10.3390/jcm12041552)
Supplement: Supplementary file 1 [file jcm-12-01552-s001.zip › jcm-2090048-supplementary/table S1.pdf]

Table S1. Precise measurement estimates with reference to figure 4. BCVA, best corrected visual acuity; CCT, central corneal thickness; GSU, grayscale units; ECD, endothelial cell density; preop, preoperatively; mo, month; Std.Dev, standard deviation.

	Mean	Confidence -95,00%	Confidence 95,00%	Median	Minimal	Maximal	Std.Dev.
BCVA_preop	0,046641	0,020964	0,072318	0,02	0,001	0,4	0,07921
BCVA_1mo	0,348462	0,279875	0,417048	0,3	0,03	0,9	0,21158
BCVA_3mo	0,44359	0,366378	0,520802	0,4	0,1	1	0,238189
BCVA_6mo	0,566667	0,483161	0,650172	0,6	0,1	1,2	0,257604
BCVA_12mo	0,617949	0,541338	0,694559	0,7	0,1	1	0,236334
BCVA_18mo	0,684615	0,613269	0,755962	0,7	0,1	1	0,220094
BCVA_24mo	0,705128	0,630779	0,779477	0,7	0,1	1	0,229357
CCT_preop	962,7179	891,1961	1034,240	971,0000	638,0000	1740,000	220,6357
CCT_1mo	598,5641	576,7531	620,375	602,0000	461,0000	766,000	67,2841
CCT_3mo	573,4103	553,1429	593,678	577,0000	450,0000	707,000	62,5223
CCT_6mo	602,6923	578,1054	627,279	602,0000	432,0000	847,000	75,8475
CCT_12mo	634,9744	608,7374	661,211	629,0000	442,0000	895,000	80,9378
CCT_18mo	675,0256	651,8896	698,162	682,0000	548,0000	875,000	71,3717
CCT_24mo	682,3333	660,8886	703,778	687,0000	566,0000	894,000	66,1543
GSU_preop	44,00603	38,64045	49,3716	42,77	16,575	80,875	16,55211
GSU_1mo	29,90269	25,79352	34,01187	27,01	12,05	60,775	12,67628
GSU_3mo	22,65679	19,53697	25,77662	20,25	11,33	50,82	9,62427
GSU_6mo	20,06885	17,4532	22,68449	17,39	11,02	44,6	8,06893
GSU_12mo	18,0659	15,74848	20,38331	15,69	10,1	40,025	7,14894
GSU_18mo	16,09218	14,46715	17,71721	14,87	10,7	34,95	5,01301
GSU_24mo	15,55051	14,05451	17,04652	14,55	9,725	32,49	4,61498
ECD_preop	2587,436	2496,083	2678,788	2557	2150	3194	281,8111
ECD_1mo	2333,026	2246,755	2419,296	2341	1850	2737	266,1346
ECD_3mo	2122,462	2036,129	2208,794	2100	1692	2689	266,3257
ECD_6mo	1889	1814,241	1963,759	1844	1541	2413	230,6234
ECD_12mo	1806,462	1730,103	1882,82	1762	1404	2398	235,5551
ECD_18mo	1719,487	1651,027	1787,948	1692	1382	2217	211,1916
ECD_24mo	1630,795	1563,731	1697,859	1607	1296	2095	206,8834
